# Effect of Radiographic Contrast Media on the Spectrin/Band3-Network of the Membrane Skeleton of Erythrocytes

**DOI:** 10.1371/journal.pone.0089512

**Published:** 2014-02-24

**Authors:** Ralf-Peter Franke, Tim Scharnweber, Rosemarie Fuhrmann, Folker Wenzel, Anne Krüger, Christof Mrowietz, Friedrich Jung

**Affiliations:** 1 University of Ulm, ZBMT, Department of Biomaterials, Ulm, Germany; 2 Institute for Biological Interfaces, Karlsruhe Institute of Technology (KIT) Karlsruhe, Germany; 3 Institute for Transplantation Diagnostics and Cell Therapeutics, Medical Center of University Düsseldorf, Germany; 4 Institute of Biomaterial Science and Berlin-Brandenburg Center for Regenerative Therapies, Helmholtz-Zentrum Geesthacht, Teltow, Germany; 5 Institute for Heart and Circulation Research, Hamburg, Germany; Emory University School of Medicine, United States of America

## Abstract

The membrane of red blood cells consists of a phospholipid bilayer with embedded membrane proteins and is associated on the cytoplasmatic side with a network of proteins, the membrane skeleton. Band3 has an important role as centre of the functional complexes e.g. gas exchange complex and as element of attachment for the membrane skeleton maintaining membrane stability and flexibility. Up to now it is unclear if band3 is involved in the morphology change of red blood cells after contact with radiographic contrast media. The study revealed for the first time that Iopromide induced markedly more severe alterations of the membrane skeleton compared to Iodixanol whose effects were similar to erythrocytes suspended in autologous plasma. A remarkable clustering of band3 was found associated with an accumulation of band3 in spicules and also a sequestration of band3 to the extracellular space. This was evidently accompanied by a gross reduction of functional band3 complexes combined with a dissociation of spectrin from band3 leading to a loss of homogeneity of the spectrin network. It could be demonstrated for the first time that RCM not only induced echinocyte formation but also exocytosis of particles at least coated with band3.

## Introduction

Radiographic contrast media (RCM) are widely used to visualize arteries e.g. in interventional cardiology. Their effects on blood vessels should be minimal [1] so that the object of the measurement – blood cells and vessels - are not influenced by the RCM molecules. However, all RCMs exhibit a more or less strong effect on endothelial cells [2]–[5] as well as on erythrocytes [6]–[14]. Up to now it is unclear which organelles or parts of the cells are effected by RCM. In a former study it was shown that RCM did not attach to or intercalate in the erythrocyte membrane and thus effect echinocytec formation as was discussed for a long time [6], [15]. There is a lot of evidence that echinocyte formation is not possible without participation of the membrane cytoskeleton. Reinhart et al. could show that the presence of spectrin is essential for echinocyte formation [16]. In addition, Becker & Lux could show that dysfunctional band3 resulted clinically in hereditary spherocytosis [17]. Therefore, it can be speculated that both constituents of the membrane cytoskeleton might interact and effect echinocyte formation what has never been shown so far.

The erythrocyte membrane consists of a phospholipid bilayer with embedded membrane proteins and is associated on the cytoplasmatic side with a network of proteins, the membrane cytoskeleton [18]. The bicarbonate/chloride exchanger band3 (Anion Exchanger 1, AE1) is the most abundant protein in the erythrocyte membrane [19]. It has an important role e.g. in gas exchange, senescence and removal of cells from the circulation, anchoring motifs for the glycolytic enzymes (GE) and functions as a point of attachment for the cytoskeleton maintaining the mechanical and osmotic properties of the erythrocyte [20], [21]. Band3 is found in three distinct protein complexes associated with the erythrocyte membrane: an ankyrin-dependent tetrameric band3 complex, a dimeric band3 complex bound to the protein 4.1-glycophorin C junctional complex and freely diffusing dimeric band3 complexes [22], [23].

It appears that an intact cytoskeleton is vital for normal cell shape. Defects or deficiencies in band3 were described to lead to a decrease in cohesion between the lipid bilayer and membrane skeleton resulting in loss of membrane surface area and a pathology termed hereditary spherocytosis [24]. Also in some haemolytic anemias, the erythrocytes can not only become spherical but are also extremely fragile, implying that the membrane skeleton furnishes stability and elasticity to the cells [17], [25]–[29]. It was shown that in mice with a knock out of band3 immediate post-natal death of most pups coincided with a high fragility of the erythrocyte membrane [30].

Reinhart et al. reported that echinocytes did not occur in spectrin-deficient knock-out mice [16]. Therefore, it can be assumed that spectrin is also a key element of erythrocytic shape changes. Also the link between the membrane skeleton and the membrane seems to be important for normal shape as rupture of the interaction of the linking proteins prevents any shape changes and reduces deformability of red cell membranes [31], [32].

It was reported that under physiological conditions the spectrin net is linked to band3 via multiple high affinity protein-protein interactions (e.g. ankyrin, adducin, protein 4.1, glycophorin [20]).

It is unknown whether the distances change between binding partners, especially spectrin and band3, during echinocytic shape changes. Closely spaced elements like band3 (stained red) or spectrin (stained green) will be subjected to a colour shift from red/green to yellow when these two elements approach one another and become co-localized. Therefore, a co-localization study was performed based on immuno-cytochemical double staining of erythrocytes. This would allow to prove whether RCM induce intracellular effects mediated by spectrin and band3 and whether both RCM act in the same way.

## Materials and Methods

The purpose of the investigation was to examine whether radiographic contrast media provoked echinocyte formations are accompanied by a reorganisation of band3 and/or spectrin filaments.

### Ethics Statement

The study protocol conformed to the ethical guidelines of the 1975 Declaration of Helsinki as reflected in a priori approval by the institutional review committee of the Medical Faculty of the Heinrich Heine University Düsseldorf (registry number: 3522). Written informed consent was obtained from each subject before entry into the study.

### Radiographic Contrast Media

Two radiographic contrast media with variant Iodine concentrations and approved for intra-arterial application were examined: (Iodixanol 320 mg Iodine/ml, GE Healthcare, München, Germany; Iopromide 370 mg Iodine/ml Bayer/Schering, Berlin, Germany).

### Blood Collection

Venous blood (20 ml) was collected in a standardized manner from the cubital veins of n = 6 healthy adults anticoagulated with potassium EDTA according to actual International guidelines [33]. The samples were stored in sealed polystyrene tubes.

### Sample Processing

Immediately after sampling, plasma and erythrocytes were separated by centrifugation (500 g, 5 min). Plasma was harvested and the plasma/radiographic contrast media mixtures required for suspension of the erythrocytes were prepared. Iodixanol or Iopromide in the concentration of 30% v/v were added to the plasma. Then, the red blood cells – without the buffy coat - were suspended in autologous plasma (control group) or in the RCM/plasma mixtures and incubated for 5 minutes at 37°C.

### Staining of Components of the Membrane Cytoskeleton

After the incubation of erythrocytes in autologous plasma or in different RCM/plasma mixtures (30% v/v Iodixanol or Iopromide, respectively), conventional blood smears were prepared on glass substrates and air dried. For each of the 3 groups 18 slides (3 from every donor) were layered with cells.

In the follow up the air dried samples were postfixed in 2% paraformaldehyde. After short rinsing in isotonic phosphate buffered saline (PBS ) at room temperature the samples were transferred into cold acetone (−20°C) for 2 minutes to render the cell membranes permeable for the antibodies. Components of the membrane cytoskeleton were double stained in consecutive steps to display the distribution of the components in the cytoskeleton. The components were stained either in red (band3; first antibody: SLC4A1 rabbit anti human polyclonal antibody (Biozol, Eching, Germany; dilution 1∶50 in PBS), second antibody: anti rabbit IgG TRITC conjugated (dilution 1∶30 in PBS)) or in green (spectrin; first antibody: mouse anti human spectrin IgG (dilution 1∶30 in PBS), second antibody: anti mouse IgG FITC conjugated (dilution 1∶30 in PBS)).

In case of a very close proximity of actin and spectrin, confocal laser scanning microscopy allows to visualise a merger of the colours red and green resulting in a colour shift towards yellow colour tones. Vice versa, if there should be a dissociation of earlier closely spaced components then there would be a colour shift from yellow coloration to red and green [34], [35]. The cells were visualized using confocal laser scanning microscopy at a primary magnification of 1∶63 (TCS SP5, Leica, Wetzlar, Germany). The appearance of pixels of preselected yellow tones in the images was quantified using an image analysis system (MacIntosh, Intelcore I7 with Adobe Photoshop, extended version). Those yellow colour tones (merger of green and red colours) were selected which were found in the roots of exocytotic-like protrusions from erythrocytes [36], [37]. 10 samples per group were examined and 4 fields of view per sample with more than 200 cells were analyzed and the mean numbers of pre-selected yellow tone pixels and the number of red dots (condensed band 3) were quantified.

For all samples mean value and standard deviation are given. For multi-sample comparisons a variance analysis for repeated measurements was performed. To isolate the group or groups that differed from the others, Student-Newman-Keuls test for paired comparisons was used. A probability value of less than 0.05 was accepted as significant.

## Results

**Figure 1:**
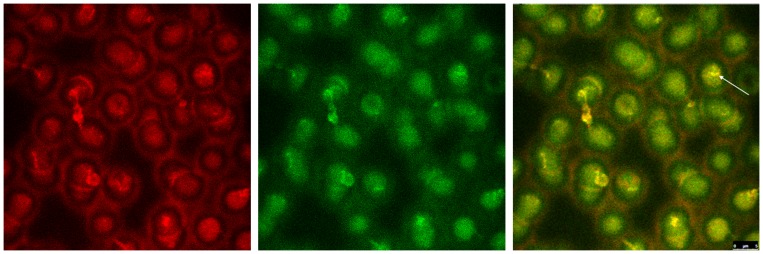
Double stained erythrocytes in autologous plasma. Band3: left image in red; spectrin: middle image in green; right image merger of red (band3) and green (spectrin). Co-localized structures are coloured in yellow tones; (primary magnification 1∶63; zoom factor 1∶2.1).

Figure 1 shows double stained erythrocytes suspended in autologous plasma in the red or the green channel of the confocal laser scanning microscope.

Discocytes revealed more band3 stain in central cell parts compared to the cell periphery. In erythrocyte aggregates the stain was enhanced in contact regions of the cells. Differing from band3 stain the spectrin stain was distributed over the cells more homogenously and there was no enhancement of spectrin stain in contact regions of aggregated erythrocytes.

The merger of red and green colour channels shows a yellow shift in a few cases (see arrow) where band3 and spectrin were co-localized. On an average 40.5±5.5 yellow pixels per field of view were found.

### Incubation of Erythrocytes in an Iodixanol/plasma-mixture (30% v/v)

Most erythrocytes were found as single cells, a few in the majority non-branched linear aggregates (rouleaux) were observed (more or less comparable to erythrocytes in autologous plasma).

**Figure 2:**
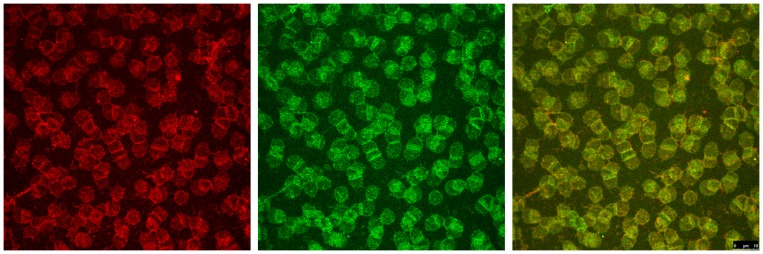
Double stained erythrocytes after incubation in plasma supplemented with Iodixanol320 (30% v/v). Band3 is stained in red (left image), spectrin in green (middle image). The merger of both colour channels is presented in the right image with co-localized structures coloured in yellow tones (30% v/v; primary magnification 1∶63; zoom factor 1∶1).

Figure 2 shows erythrocytes which were suspended in a Iodixanol/plasma-mixture (30% v/v). There were only few cells showing echinocytic shape change. Cells with spicules showed increased amounts of stained band3 in these spicules. At the same time less spectrin stain was found there indicating that spectrin did not seem to enter the spicules (therefore, in the spectrin stain it is difficult to identify echinocytes).

However, other echinocytes with well rounded protrusions exhibited spectrin staining. In the few linearly aggregated erythrocytes (rouleaux) the green stain was enhanced in the contacting regions. It can be assumed that in these contacting regions spectrin was concentrated hinting to a condensation of the spectrin network.

Compared to cells in autologous plasma the distribution of band3 and spectrin in cells suspended in Iodixanol/plasma-mixture was similar but the grain-like pattern of red-green coloured areas (merger of red and green channels in **Figure 2**) was a bit coarser hinting to a slight condensation of band3 and of spectrin where a tendency to condense seemed to be a bit stronger in band3.

After incubation of erythrocytes in a Iodixanol/plasma-mixture an increase in the number of pixels with yellow tone occurred (83.0±53.3 pixels on an average; p<0.05). The co-localization associated yellow colour tones appeared at the periphery of these erythrocytes incubated in Iodixanol/plasma-mixture and not in central parts as those which were found in erythrocytes incubated in autologous plasma.

### Incubation of Erythrocytes in a Iopromide/plasma-mixture (30% v/v)

**Figure 3:**
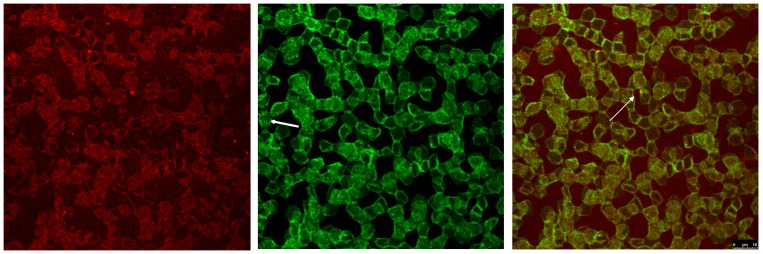
Double staining of erythrocytes after incubation in plasma supplemented with Iopromide370 (30% v/v). Band3 (left image) is coloured in red; spectrin (middle image) in green. The merger of red (band3) and green (spectrin) channels is presented in the right image with co-localized structures in yellow tones (primary magnification 1∶63; zoom factor 1∶1). Arrow in the middle image points at a cell with condensed spectrin bands in the central part of the cell, arrow in the right image points at condensed band3 (red dot) in spicule tip.

**Figure 4:**
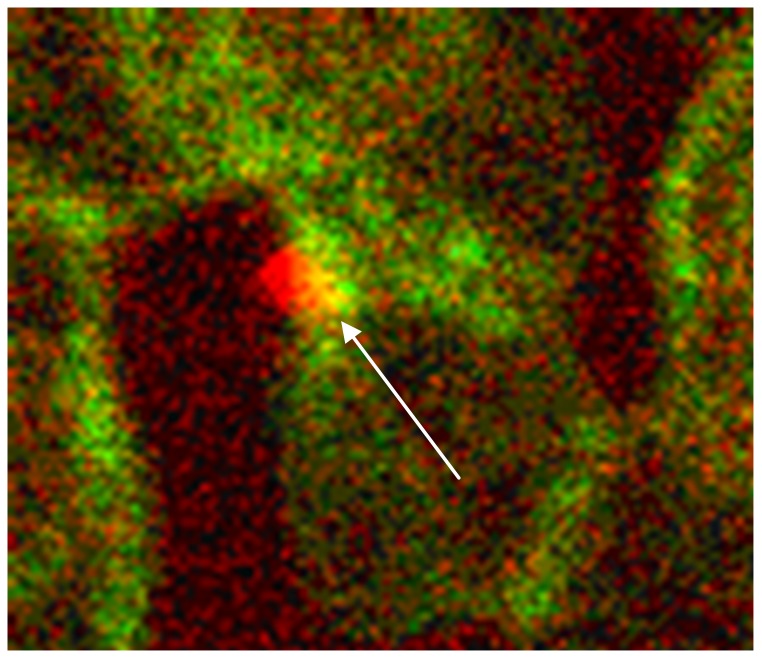
Co-localization of spectrin and band3 at the root of a spicule. Band3 is coloured in red, spectrin in green. The co-localization of both proteins at the root of a spicule (revealed by the yellow colour shift) is marked by an arrow (primary magnification 1∶63; zoom factor 1∶15).

Figure 3 reveals that band3 stain was arranged in a rectangular fashion unlike the arrangement of band3 stain in erythrocytes suspended in autologous plasma or in Iodixanol/plasma-mixture. The formation of spicules can be observed in band3 stained erythrocytes showing a clear enhancement of band3 in these spicules (arrow in Figure 3 merged image), especially in very pointed ones, often revealing band3 in the tip of the spicule (arrow in Figure 4).

Compared to erythrocytes in autologous plasma or in a Iodixanol/plasma-mixture a homogenous spectrin stain was no longer found in erythrocytes suspended in a Iopromide/plasma-mixture (Figure 3 middle). Most of the cells were found in linear branched or un-branched or in non-linear branched aggregates (rosettes or cross-like aggregates) where the spectrin stain was mainly concentrated at the border regions of contacting cells and often running through central parts of cells as a fibre-like network (arrow in Figure 3 middle). One of the most obvious findings and most clear to recognize was the rectangular arrangement of the spectrin network – completely different from the homogenous distribution and more or less round arrangement of spectrin in erythrocytes suspended in autologous plasma or in a Iodixanol/plasma-mixture.

**Figure 5:**
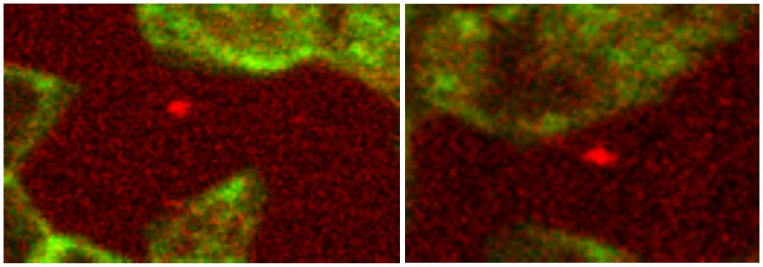
Condensed band3 outside of erythrocytes. Details from figure 3 right (image electronically magnified): condensed band3 stain outside of erythrocytes (left image) or loosely connected to the erythrocyte membrane (right image) (primary magnification 1∶63; zoom factor 1∶10).

The merger of red (band3) and green (spectrin) channels showed that co-localizations were rarely seen (44.0±34.2 pixels on an average) and that there was a multitude of relatively fine grained red dots revealing a concentration of band3 in echinocytic spicule tips (arrows in Figure 3 merger of red and green channels, Figure 4) or outside of cells possibly extruded from the spicule tips (see Figure 5).

Figure 4 shows an erythrocyte in detail with condensed band3 in spicule tip marked by an arrow in Figure 3. Co-localizations of spectrin and band3 (see yellow colour in Figure 4 marked by arrow) appeared almost exclusively at the roots of the spicules and not in central parts of erythrocytes (as in cells incubated in autologous plasma).

Figure 5 demonstrates for the first time that particles at least coated with band3 were found outside of erythrocytes and gives evidence that not only echinocyte formation with spicules occurred after RCM contact but also exocytosis with the export of band3 from erythrocytes.

### Quantitative Image Analysis

An image-analysis showed that the appearance of pixels with selected yellow tones in red/green merged images, due to a co-localization of band3 (red stained structures) and spectrin (green stained structures), differed significantly between the three groups (Kruskal Wallis test: p = 0.003). Using a post-hoc analysis (Tukey tests) it could be shown, that cells in a Iodixanol/plasma mixture showed significantly more co-localized spectrin and band3 (83.9±53.3 yellow tone pixels) than cells suspended in autologous plasma (40.4±5.5 yellow pixels; p<0.05) or cells suspended in a Iopromid/plasma mixture (44.4±34.2 pixels; p<0.05).

Also the number of condensed band3 dots inside of erythrocytes was counted. In cells suspended in autologous plasma a mean number of 5.5±1.6 condensed band3 dots was found, in cells suspended in a Iodixanol/plasma mixture 27.5±4.9 and in cells suspended in a Iopromide/plasma mixture 16.8±5.1 condensed band3 dots (p<0.001) were found. The number of dots in the Iodixanol/plasma mixture differed significantly from the number in cells in autologous plasma and as well from the number in Iopromide/plasma mixture (p<0.05 each).

In a third analysis the numbers of band3 particles outside of erythrocytes were counted. Here, the three groups also differed significantly (p<0.001). After suspending erythrocytes in a Iopromide/plasma mixture 6.7±1.4 band3 particles were found, after suspending erythrocytes in a Iodixanol/plasma mixture 1.3±1.2 particles and in cells suspended in autologous plasma 0.17±0.4 particles were found. The number of band3 particles in the Iopromide/plasma mixture differed significantly from the number of such particles in autologous plasma and as well from the number in a Iodixanol/plasma mixture (p<0.05 each).

## Discussion

The study revealed that the shape changes or the aggregation of erythrocytes in contact with radiographic contrast media (RCM) clearly coincided with changes in the structure of the membrane skeleton depending on the type of RCM used. This was most clearly demonstrated by the abolishment of the homogenous spectrin distribution with a relocation of spectrin primarily to the rim of erythrocytes and accompanied by appearance of some thick bundles of spectrin crossing central parts of cells suspended in a Iopromide/plasma-mixture but not in a Iodixanol/plasma-mixture (in accordance with the results from a former study [36]). Another remarkable finding was the formation of rosette-like erythrocyte aggregates only in Iopromide/plasma-mixtures. This seems to imply an activation of complement according to Luginbühl [38], which was found to occur *in vivo* after application of hyperosmolar RCM (+70.2%, p<0.00001) but only weakly (+9%, p<0.026) [39] or not at all [40] after application of isoosmolar RCM. This is in good agreement with former studies describing a weak increase in linear erythrocyte aggregates but not the formation of rosettes [9], [36], [41].

Remarkable differences appeared with respect to the formation of protrusions and spicules which contained band3. Small amounts of protrusions and spicules were demonstrated in erythrocytes suspended in autologous plasma or in a Iodixanol/plasma-mixture (slightly more than in autologous plasma). Considerably more protrusions and spicules were found after suspension of erythrocytes in a Iopromide/plasma-mixture. These results correlate very well with findings in trans-illuminated microscopical studies [10], [42]. It has to be kept in mind that in the study reported here the silhouettes of the membrane cytoskeleton were displayed and not the shapes of the cell membrane.

**Figure 6:**
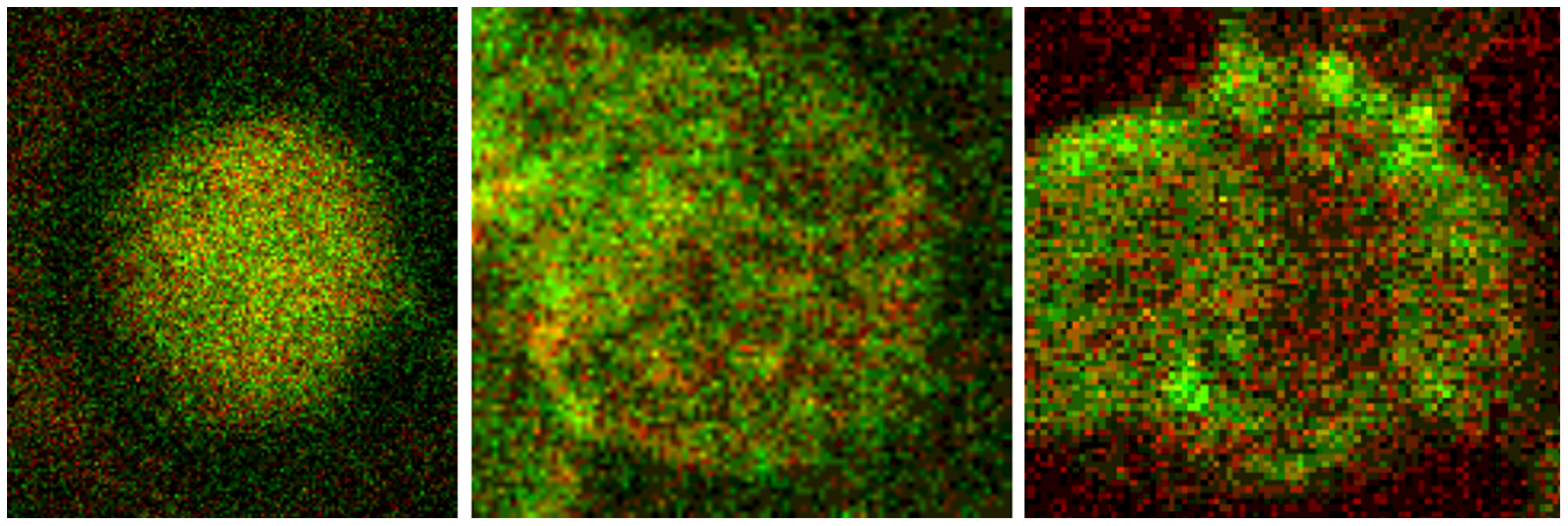
Merger of red (band3) and green (spectrin) channels after examination in a confocal laser scanning microscope. Double stained erythrocytes with antibodies against band3 (red) and spectrin (green, details from **Figures 1** **,** **2** **,** **3**). Left: erythrocyte suspended in autologous plasma, middle: erythrocyte suspended in a Iodixanol/plasma-mixture (30% v/v), right: erythrocyte suspended in a Iopromide/plasma-mixture (30% v/v; primary magnification 1∶63; zoom factor 1∶10).

The major structural member of the erythrocytic membrane skeletal network is the spectrin tetramer [43]. In this study changes in the membrane skeleton of erythrocytes were characterized (see Figure 6) by a transition from a homogenous distribution of the spectrin network (cells in autologous plasma, or in a Iodixanol/plasma-mixture) to the generation of spectrin bundles mostly concentrated at the cell rim and sometimes found also in central parts of the erythrocytes (cells in a Iopromide/plasma-mixture). This transition was described to be caused by disruption of the ankyrin-band3 binding [44], coinciding with a loss of nearness of spectrin dimers necessary for their self-association [45]. The tetrameric state of spectrin is assumed to be essential in order to maintain the normal membrane mechanical stability [13], [43], [46]. Shear elasticity of the red cell membrane was reported to be regulated by extension of spectrin tetramers and a reversible unfolding of constituent triple helical repeats [18]. The adducin-band3-spectrin bridge - more recently described [20] - probably contributes also to membrane stability.

Figure 6 left shows a representative image of erythrocytes suspended in autologous plasma displaying a more or less homogeneous distribution of band3 and spectrin. Erythrocytes suspended in Iodixanol/plasma mixtures (middle) showed a little less homogeneous distribution of band3 and spectrin with a few small aggregates of spectrin found in the majority in central parts of the cells. Yellow tone areas due to co-localization of band3 and spectrin appeared in the periphery. The right image shows an erythrocyte suspended in a Iopromide/plasma-mixture. The most important aspect is the arrangement of few very coarse aggregates of spectrin almost completely found at the periphery of cells.

**Figure 7:**
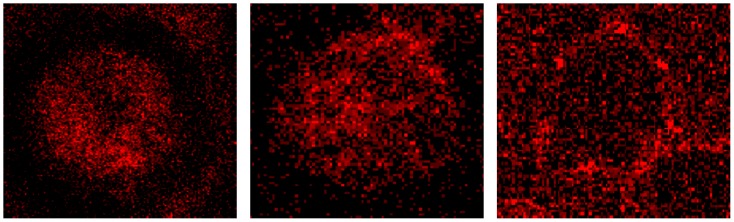
Representative erythrocytes immunologically stained with antibodies against band3 (details from Figures 1, 2, 3). Left: erythrocyte suspended in autologous plasma, middle: erythrocyte suspended in a Iodixanol/plasma mixture (30% v/v), right: erythrocyte suspended in a Iopromide/plasma mixture (30% v/v; primary magnification 1∶63; zoom factor 1∶10).

Erythrocytes in autologous plasma displayed a more or less homogeneous distribution of fine-grained band3 (Figure 7, left). Erythrocytes in Iodixanol/plasma-mixture showed a band3 distribution which was less homogeneous with a few coarser band3 aggregates in central parts of the cell as well as in the periphery. Erythrocytes in Iopromide/plasma-mixture displayed an inhomogeneous pattern of band3 with almost no band3 aggregates in central parts and very few coarse band3 aggregates concentrated at the cell rim.

**Figure 8:**
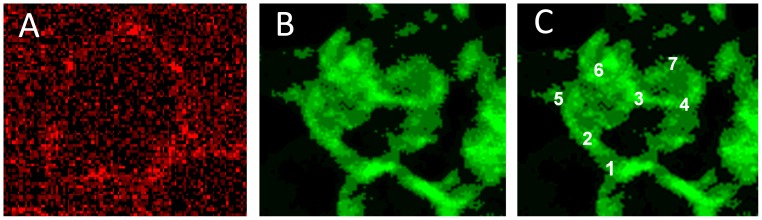
Band3 and spectrin structures after Iopromide contact. Band3 aggregates (A) and box-like spectrin configuration (B, C) of single erythrocytes after contact with Iopromide (primary magnification 1∶63; zoom factor 1∶10).

In a very recent study Franke et al. [36] reported that upon Iopromide addition the spectrin net usually multiply linked to the erythrocyte membrane abandoned its well- rounded shape near the cell membrane now forming a box-like structure almost rectangular and linked to the erythrocyte membrane via very few – about 8 to 10– junctional elements [36], which affords the dissociation of the vast majority of connections between the spectrin network and the membrane. Such a box-like structure of the spectrin net after addition of Iopromide to the plasma is shown in Figure 8B and C with numbers from 1–7 depicting the corners of such a box-like structured spectrin configuration (Figure 8C; where corner number 8 is hidden behind number 3).

It might be hypothesized that the very few coarse band3 aggregates shown in Figure 7C and Figure 8A are the junctional elements binding the rectangular box-like spectrin configuration to the erythrocyte membrane.

Band3 is described to constitute a centre organized into complexes for gas transport [47]–[50], for cation efflux pumping (anion exchanger) [51]–[53], for glycolysis [54], [55], for the control of cell volume [56] and of erythrocyte life span (senescence) [57], where most probably further complexes exist to cope with mechanical deformation [32], [58], and haemostatic stimuli [59]. The drastic reorganization of the band3 - spectrin network effected by suspension of erythrocytes in a Iopromide/plasma-mixture coincided with a strong alteration of the spectrin network and a clustering of band3 in very few highly condensed centres at the rim of the cells. This is most probably accompanied by a strong increase in membrane stiffness (contributing to microcirculatory disorders shown in patients with coronary artery disease after Iopromide application during coronary angiography [60], [61]). Also a strong decrease of centres for gas transport can be expected contributing to hypo-oxygenation of the tissue as was shown after Iopromide application in the myocardium of pigs [62] but not after Iodixanol application [63]. The clustering of band3 is assumed to lead to a shortening of the lifespan and removal of erythrocytes by offering senescence signals.

This study revealed for the first time a remarkable loss of band3 after contact of erythrocytes with Iopromide demonstrated by a sequestration of band3 probably out of the tips of the spicules giving evidence that the echinocyte formation provoked by Iopromide seems to be associated with an exocytotic-like process – a mechanism well known from somatic cells containing nuclei [64], [65], but up to now not described in erythrocytes. As band3 is a key element of binding the membrane cytoskeleton to the erythrocyte membrane, the loss of band3 could be associated with an irreversible reduction of membrane cytoskeleton binding potential. This irreversible reduction might be the reason that the echinocyte formation was shown to be reversible only for a certain fraction of echinocytes after their resuspension in autologous plasma [66]. It might be hypothesized that particularly older erythrocytes – with less cell volume [67], and decreases in haemoglobin [67] and cell function [68] – could be the prime targets.

### Conclusion

Differences in the formation of echinocytes after their suspension in RCM/plasma-mixtures were reported earlier [9]–[13], [41] and are in good agreement with the results from this study. This co-localization study, however, goes far beyond as differences exerted by Iodixanol versus Iopromide in the formation of protrusions and spicules and of echinocytes could be attributed to subcellular phenomena, however, in different ways. It could be demonstrated for the first time that Iopromide induced echinocyte formation coinciding with a more or less rectangularly arranged spectrin configuration mainly concentrated at the border regions of contacting cells and often running through central parts of cells as a fibre-like network – completely different from the homogenous spectrin distribution of cells in autologous plasma or in a Iodixanol/plasma-mixture. In addition, also a process resembling exocytosis of particles at least coated with band3 could be demonstrated after incubation of erythrocytes in Iopromide/plasma-mixture.

Iopromide, evidently, induced different and markedly more severe alterations of the membrane skeleton compared to Iodixanol whose effects were similar to erythrocytes suspended in autologous plasma.
